# Hospitalizations and In-Hospital Fatality Among Influenza Patients in the Pre-Pandemic and COVID-19 Pandemic Periods

**DOI:** 10.3390/jcm14061785

**Published:** 2025-03-07

**Authors:** Krzysztof Kanecki, Katarzyna Lewtak, Piotr Tyszko, Paweł Goryński, Michał Rząd, Katarzyna Okręglicka, Aneta Nitsch-Osuch

**Affiliations:** 1 Department of Social Medicine and Public Health, Medical University of Warsaw, 00-106 Warsaw, Poland; krzysztof.kanecki@wum.edu.pl (K.K.); piotr.tyszko@wum.edu.pl (P.T.); michal.rzad@wum.edu.pl (M.R.); katarzyna.okreglicka@wum.edu.pl (K.O.); aneta.nitsch-osuch@wum.edu.pl (A.N.-O.); 2 Institute of Rural Health in Lublin, 20-950 Lublin, Poland; 3 School of Public Health, Centre of Postgraduate Medical Education, 01-826 Warsaw, Poland; pgorynski@cmkp.edu.pl; 4 Department of Internal Medicine, Pneumonology, Allergology, Clinical Immunology and Rare Diseases, Military Institute of Medicine—National Research Institute, 04-141 Warsaw, Poland

**Keywords:** in-hospital fatality, hospitalization incidence, influenza, COVID-19, vaccine-preventable disease

## Abstract

**Background/Objectives**: Despite being a vaccine-preventable disease, influenza continues to pose a significant global health challenge, with clinical severity increasing at the extremes of age. This study aimed to describe influenza hospitalizations and hospital-related fatality rates in Poland in the pre-pandemic and COVID-19 periods. **Methods**: This retrospective population-based study included 39,604 hospital admissions with a diagnosis of influenza in Poland. Data were extracted from the National General Hospital Morbidity Study conducted by the National Institute of Public Health for the period 2016–2022. **Results**: Based on the hospital registry and data on the general population, an upward trend in hospitalization rates was observed in 2016–2020. In subsequent years, the lowest hospitalization rates were observed in 2021 and the highest ones in 2022, accounting for 1.3 and 30.3 hospitalizations per 100,000, respectively. Two hospitalization peaks were observed, one for children 0–5 and the other for adults 65–70 years of age. After the COVID-19 outbreak in Poland, an increased percentage of hospitalizations was observed in children and adolescents, and a decreased frequency was found in the elderly. The overall hospitalization fatality rate during the study period accounted for 2.9%, and the authors observed a decrease in the in-hospital fatality rate after the COVID-19 outbreak compared to the pre-pandemic period (3.4% vs. 2.1%, *p* < 0.001). **Conclusions**: This study highlights recent trends in influenza hospitalizations and in-hospital mortality before and during the COVID-19 pandemic in Poland, providing important data for optimizing prevention strategies and serving as a foundation for global comparative analyses.

## 1. Introduction

The majority of influenza virus infections are mild and self-limiting, with only a small fraction developing into severe illness requiring hospitalization. During epidemics, school-aged children typically exhibit the highest rates of influenza virus infections, whereas the severity of illness tends to increase in the very young and the elderly patients [1]. Older adults often present atypically, and they show complications of influenza infection, such as chronic obstructive pulmonary disease and congestive heart failure exacerbations [2]. The groups at high risk of poor outcomes are the elderly, the very young, pregnant women, and those with chronic health conditions. Adults aged 18–64 years with underlying comorbidities are at an increased risk of influenza-related hospitalizations, ICU admission, and mortality as compared to otherwise healthy individuals [3]. A higher risk of experiencing serious complications from influenza is observed in older adults. It is often ascribed to the weakening of the immune system with age, and despite widespread vaccination programs, 90% of influenza-related deaths occur in older adults. Common chronic conditions contribute not only to the loss of immune protection after vaccination and increase the risk for severe outcomes of influenza but also increase the long-term consequences following hospitalization [4]. Despite being a vaccine-preventable disease, influenza causes approximately 3–5 million cases of severe illness and about 290,000–650,000 deaths worldwide, which occur primarily in persons aged 65 years and older [5]. One meta-analysis reported that globally, influenza viruses accounted for over 5 million hospitalization cases per year, and influenza viruses contributed to severe course of illness and hospitalizations among both younger and older adults [6].

A factor that can undoubtedly reduce the risk of hospitalization and overall and in-hospital mortality among older people is the use of influenza vaccinations. Immunosenescence was found to be the reason behind the generally poor vaccine effectiveness in the elderly [7]. In a study conducted by Behrouzi et al., vaccination was linked to a 34% reduction in the risk of major adverse cardiovascular events, with individuals who recently experienced acute coronary syndrome exhibiting a 45% lower risk [8]. In a systematic review and meta-analysis of data based on randomized controlled trials, the influenza vaccine was not significantly associated with lower all-cause mortality, cardiovascular death, or cardiovascular disease hospitalization. However, a statistically significant reduction in all-cause hospitalizations was observed [9]. The COVID-19 pandemic has changed healthcare usage patterns, affecting the organization of influenza vaccinations and the hospitalization of influenza patients. On 4 March 2020, Poland detected its first COVID-19 case, marking the beginning of the first wave of infections. Immediately afterward, to mitigate public health risks, restrictions were introduced, including limits on non-urgent medical services at all levels of healthcare. As case numbers declined, healthcare access was gradually restored. In 2020, hospital treatments decreased by nearly 25%, outpatient specialist services by 19%, and primary healthcare services by almost 11%. The number of patients declined proportionally. By 2021, the availability of healthcare services returned to near pre-pandemic levels [10]. As of 2 February 2025, Poland recorded 6,773,561 SARS-CoV-2 infections (6,466,890 by the end of 2022) and 120,977 reported COVID-19 deaths (118,533 by the end of 2022) [11,12,13].

Hospitalization and mortality rates in older adults may be related to influenza, but similar risks have been observed in other viral infectious diseases. In one systematic review and meta-analysis, hospitalization and mortality rates were similar for RSV and influenza in older adults [14].

Hospital discharge databases could serve as an important source of information on disease burden and the challenges faced by healthcare systems [15]. The necessity for hospitalization indicates not only illness severity or prevalence of ill health but also highlights unmet primary healthcare needs. Understanding the impact of influenza on hospitalization and the risk of hospital death based on national register of hospital morbidity may be particularly justified due to the need to obtain data for further comparative analyses of the impact of infectious diseases on hospital stays and hospital deaths. Additionally, analyzing changes in influenza hospitalizations and in-hospital mortality following the COVID-19 outbreak could enhance prevention policies and develop targeted strategies for groups most in need [16]. However, the influence of the COVID-19 pandemic on influenza hospitalizations in Poland remains insufficiently studied.

The aim of the study was to describe influenza hospitalizations and hospital-related fatality rates in Poland in the pre-pandemic and COVID-19 periods.

## 2. Materials and Methods

### 2.1. Study Design and Data Source

This was a retrospective, population-based study on influenza hospitalizations in Poland from 2016 to 2022, using the national hospital discharge database, the Nationwide General Hospital Morbidity Study (NGHMS), conducted by the National Institute of Public Health NIH-NRI. The NGHMS collects anonymized data on all hospitalizations in Poland, excluding psychiatric care units, including patient demographics (age, sex, and place of residence), hospital admission and discharge details (such as completing therapeutic or diagnostic processes, referrals to outpatient care or other hospitals, or patient death), and information on principal diagnoses and comorbidities, coded according to the International Classification of Diseases, 10th revision (ICD-10).

Information on the number of influenza cases in Poland, as well as on the influenza vaccination coverage, was obtained from reports on infectious disease cases published by the National Institute of Public Health NIH-NRI in Poland [13]. Data for the Polish population were collected from Statistics Poland as of 30 June for each year.

### 2.2. Variables

Influenza-related hospitalization was defined as any recorded hospital admission with an influenza diagnosis, identified by ICD-10 codes J10 (influenza due to identified influenza virus) or J11 (influenza due to unidentified influenza virus), listed as either the primary or secondary diagnosis, between 1 January 2016 and 31 December 2022.

The study was divided into two periods: the pre-pandemic period, from 1 January 2016 to 3 March 2020, and the pandemic period, which began on 4 March 2020, following the first confirmed COVID-19 case in Poland, and continued until 31 December 2022.

We included the overall number of hospitalizations due to influenza, categorized by patient age and sex. Age groups were defined as 0–4, 5–19, 20–64, and ≥65 years. The hospitalization rate was calculated using population data from Statistics Poland [17]. The in-hospital case fatality rate (CFR), representing the percentage of patients who died following hospital admission with influenza (J10-11), was calculated by dividing the number of deaths by the total number of patients with influenza-related hospitalizations.

### 2.3. Statistical Analysis

To perform most statistical analyses, Statistica (TIBCO Software Inc, Palo Alto, CA, USA) version 13) [18] and WINPEPI [19] were used. For continuous variables with normal or non-normal distribution, means and SD, or medians and IQR were computed, respectively. For nominal variables, counts and percentages were analyzed. Statistics Poland (national census) data were used as denominators [17]. Continuous variables were compared between groups using Student’s *t*-test, considering the assumption of normal distribution in sufficiently large samples in public health research following the central limit theorem [20]. The distribution of qualitative data between groups was compared using the chi-square test. Linear regression was used to assess trends. When reporting linear models, the coefficient of determination (R2) was presented. A two-sided *p*-value less than 0.05 was considered to be statistically significant.

## 3. Results

**Figure 1:**
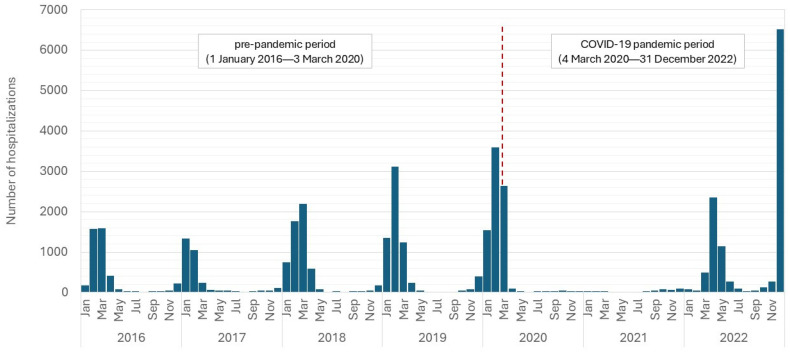
The monthly distribution of hospitalization cases (2016–2022).

From January 2016 to December 2022, a total of 39,604 hospitalizations with an influenza diagnosis were recorded in Poland. The monthly distribution of hospitalization cases is presented in Figure 1. Influenza due to an identified influenza virus (ICD-10 code: J10) accounted for 87% of all cases, while influenza due to an unidentified influenza virus (ICD-10 code: J11) was reported in 13% of the cases.

**Figure 2:**
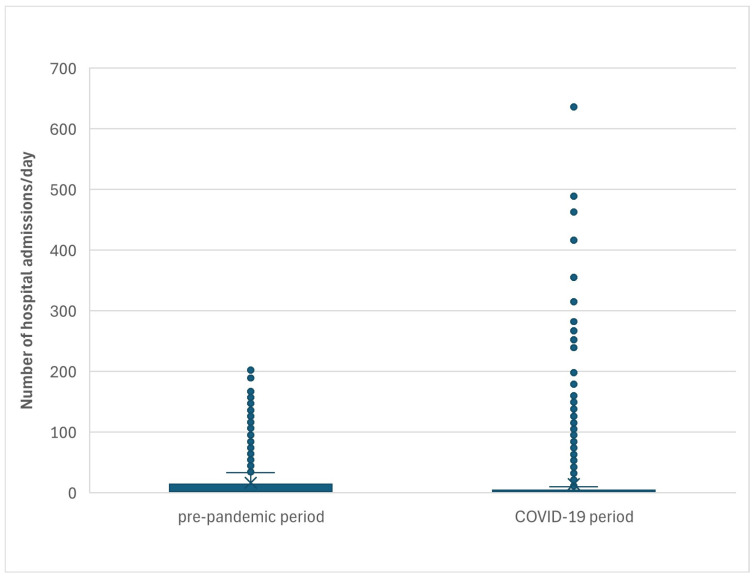
Number of hospital admissions per day in pre-pandemic period and COVID-19 pandemic period.

Throughout the analyzed period, the mean daily number of hospital admissions due to influenza was 15.5 (SD = 40.0), ranging from 0 to 636 (Figure 2). In the pre-pandemic period, the mean daily admission rate was 16.5 (SD = 31.9; range: 0–202), whereas during the COVID-19 pandemic, it decreased to 14.0 (SD = 49.7; range: 0–636). The lowest mean daily admission rate was recorded in 2021 at 1.3 (SD = 1.7; range: 0–12), while the highest was observed in 2022, reaching 31.3 (SD = 76.2; range: 0–636).

In the study group, there was a notable predominance of male patients, with 21,406 males (54.1%) and 18,197 females (45.9%), *p* < 0.001, when compared to the sex distribution in the general Polish population. Male patients’ predominance ranged from 51.2% in 2017 to 55.3% in 2016 in the study period. In one case, the sex was recorded as either male or female.

**Figure 3:**
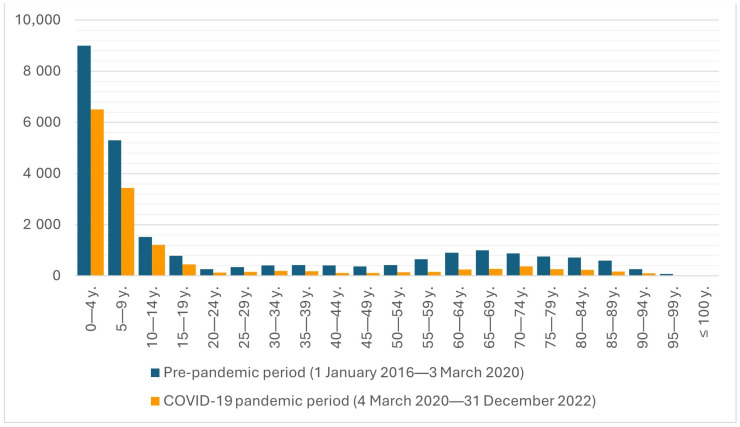
Age distribution among the study group in the pre-pandemic (1 January 2016–3 March 2020) and COVID-19 pandemic periods (4 March 2020–31 December 2022).

The age distribution of the study group in pre-pandemic and COVID-19 periods is presented in Figure 3.

As reported in Figure 3, the age distribution was similar to the two-peak distribution, with a predominance of children and adolescents in both periods. Four age groups were defined with the following ranges: 0–4, 5–19, 20–64, and 65+ years. This age differentiation was also made due to reports from other countries, where the age groups 0–4 and 65+ were mainly reported [18,19]. The age and sex comparisons in the abovementioned age range in the pre-pandemic and COVID-19 pandemic periods are presented in Table 1. According to the data in Table 1, most hospitalizations were observed in the groups of patients aged 0–4 and 5–19 years of age. Considering the pre-pandemic and pandemic periods, the most significant changes were observed in the youngest age group (0–4 years), where the proportion increased by 9.1 percentage points, rising from 35.9% before the pandemic to 45.0% during the pandemic. In contrast, the proportion of cases in all adult age groups declined.

Data on the number of influenza-related hospitalizations and the influenza hospitalization rate per 100,000 population in Poland, categorized by age group, for the years 2016–2021 are presented in Table 2.

The mean annual hospitalization incidence significantly decreased in 2021 in comparison to 2020 (21.1 vs. 1.3 per 100,000, *p* < 0.001), and it significantly and greatly increased from 2021 to 30.3 per 100,000 in 2022 (*p* < 0.001). A significant increase trend in hospitalizations was observed from 2016 to 2020 (*p* < 0.05; R2 = 0.81).

The highest hospitalization rates per 100,000 population were observed in the youngest age group (0–4 years) across both the pre-pandemic and COVID-19 pandemic periods. In the pre-pandemic years, rates ranged from 52.5 per 100,000 in 2017 to 136.4 per 100,000 in 2019, whereas during the COVID-19 pandemic, they fluctuated between 15.8 per 100,000 in 2021 and 301.0 per 100,000 in 2022.

Between 2016 and 2022, 1146 of those hospitalized died (Table 3). The overall hospitalization fatality rate during the study period accounted for 2.9%, and we observed a decrease in the in-hospital fatality rate after the COVID-19 outbreak compared to the pre-pandemic period (3.4% vs. 2.1%, *p* > 0.001). Additionally, during the pre-pandemic period, the percentage of fatal hospitalizations increased from 3.9% in 2016 to 4.5% in 2019, and then, significantly, it decreased from 2019 to 1.5% in 2022 (*p* < 0.001).

In-hospital fatality rates were the highest in older patients; however, small fluctuations were observed in fatality rates in the study period.

Table 4 summarizes all documented cases of influenza within the general population and the incidence per 100,000 population, providing an overview of the epidemiological trends of influenza in Poland from 2016 to 2022. It also includes information on influenza vaccination coverage within the general population.

## 4. Discussion

The COVID-19 pandemic began in late 2019 and swiftly became a global public health crisis, impacting the epidemiology of various respiratory viruses, including influenza [21]. The presented study is the first nationwide study in Poland to assess the impact of the COVID-19 pandemic on the epidemiology of influenza, including the severity of the course of illness resulting in the need for hospitalization and the in-hospital mortality. As in other studies, we found a significant decrease in the number of influenza infections requiring hospitalization in Poland immediately following the outbreak of the COVID-19 pandemic compared to years in the pre-pandemic era [22,23]. Nearly 40,000 hospitalizations due to influenza were recorded in Poland during the analyzed period, i.e., 2016–2022. The rate of hospitalizations per 100,000 residents before the pandemic ranged from 7.9 (2017) to 17.1 (2019) and reached its lowest level in 2021 (1.3/100,000). The changes in hospitalization rates reflect global trends related to the impact of the pandemic on influenza epidemiology, and our results are consistent with observations from other countries, where there was also a significant decline in influenza-related hospitalizations following the outbreak of the COVID-19 pandemic. In Wales, prior to the COVID-19 pandemic, annual incidence rates per 100,000 population for admissions with influenza-specific diagnosis codes ranged from 12.0 in 2015 to 78.1 in 2019. These declined to 13.5 and 3.5 in 2020 and 2021, respectively [22]. In Spain, the hospitalization rate due to influenza was 54.2 per 100,000 population, with a range from 0.5 per 100,000 in 2020–2021 to 92.9 in 2017–2018 [23]. The hospitalization rates recorded in the pre-COVID-19 era were higher than those in France, which reported 28.5 hospitalizations per 100,000 population, and Portugal, which had 11.6 cases per 100,000. However, these rates were comparable to those observed in Norway, with 48 hospitalizations per 100,000, and the United Kingdom, where the rate was 49 per 100,000 [24,25,26,27].

The highest hospitalization rates were among 0–4-year-olds, and the lowest among 20–64-year-olds. Before the pandemic, hospitalizations among the youngest were 52.5–136.4/100,000; during the pandemic, they were 15.8–301.0/100,000. For adults (20–64 years), the values were much lower—2.1–4.6/100,000 before and 0.2–4.6/100,000 during the pandemic. In the 65+ group, the rates dropped from 9.0–19.9/100,000 before the COVID-19 pandemic to 1.0–14.3/100,000 during the pandemic. A systematic review of 127 studies published between 1995 and 2020 estimating influenza-associated hospitalizations found that the overall rate of influenza-associated hospitalizations was 40.5 (95% CI: 24.3–67.4) per 100,000 people, with rates varying widely by patient age: from 224.0 (95% CI: 118.8–420.0) in children aged 0–4 years to 96.8 (95% CI = 57.0–164.3) in elderly patients aged >65 years. National hospitalization rates (across all age groups) varied widely, ranging from 11.7 (95% CI: 3.8–36.3) per 100,000 in New Zealand to 122.1 (95% CI: 41.5–358.4) per 100,000 in India (all age group estimates) [28]. In an 11-year retrospective study conducted in Italy, covering the seasons from 2008/09 to 2018/19, the majority of hospitalizations occurred among two age groups: the elderly, aged over 65 years, and young children, aged 0–4 years. The incidence rates for these groups were 86 cases per 100,000 for the elderly and 125 cases per 100,000 for young children [29]. In Spain, the estimated average annual number of hospitalizations related to influenza was 75 cases per 100,000 people during the period from 2008 to 2018. In Portugal, the estimated average was lower, at 51.5 cases per 100,000 during the same time frame. Additionally, the population aged 65 years and older in Spain reached 335.3 cases per 100,000, while in Portugal, it was 200 cases per 100,000 [25,30].

During flu seasons from 2010–11 to 2022–23 in the U.S., adults aged 65 and older consistently had the highest rates of influenza-related hospitalizations among all age groups, with children aged 0–4 years following in most seasons [31]. The decline in the median age of hospitalized influenza patients in Poland during the analyzed period is significant. It decreased from 24.3 years in the pre-pandemic period to 15.9 years during the COVID-19 pandemic. This could be attributed to an increase in hospitalizations among children and adolescents aged 0–19, whose share rose from 66.3% in the pre-pandemic period to 80.2% during the pandemic. Both in the pre-pandemic period and during the pandemic, hospitalized patients were mainly male (pre-pandemic era: 53.7%; pandemic era: 54.7%). According to previous studies, men have a higher risk of contracting influenza compared to women in all age groups during an influenza virus epidemic [32,33]. On the contrary, a survey of hospital admissions due to influenza from the Hospital Episode Statistics database in England, covering September 2016 to March 2020, showed a predominantly female demographic at 56% [34]. In a study of influenza-related hospitalizations among U.S. adults from 2016 to 2023, a slightly higher percentage of hospitalized patients were women (range: 52.9% to 56.5%) [35]. A study conducted in Spain from July 2016 to June 2021 revealed that males slightly outnumbered females among patients hospitalized for influenza, both during the pre-pandemic and pandemic seasons. For instance, in the 2017–2018 influenza season, males represented 51% of all hospitalizations, while during the 2020–2021 pandemic season, their proportion increased to 59.3% [23].

The decline in influenza cases and the need for inpatient treatment resulted from non-pharmacological interventions (NPIs) aimed at reducing the exposure and transmission of SARS-CoV-2 within the community. It is well known that many of the NPIs implemented during the COVID-19 pandemic, including lockdown, quarantine, wearing masks, social distancing, and frequent hand washing, reduce influenza infections [22,36,37,38,39]. In mid-March 2020, Poland implemented COVID-19 mitigation measures, including travel restrictions, handwashing guidelines, social distancing, and face mask mandates. Lockdowns were enforced, resulting in stay-at-home orders and school closures. An epidemic state was declared on March 23, 2020. NPIs largely contributed to the rapid decline in influenza cases, including those requiring hospitalization from April 2020, which continued until March 2022, when the restrictions (including the order to cover the mouth and nose with a mask and the obligation to maintain social distance indoors) were lifted. With the relaxation of public health measures due to COVID-19 worldwide, inter-seasonal recurrences of respiratory virus infections were observed. However, at the end of 2022, with the normalization of population movement and social activity in Poland, influenza activity increased sharply, leading to an increase in the number of cases requiring hospitalization. Among others, a similar situation occurred in China in the first months of 2023 after lifting pandemic-era restrictions [40].

In 2022, the rate of hospitalizations per 100,000 population in Poland peaked at 30.3. In France, as in our study, there were minimal values for influenza hospital admissions in the first year of the pandemic, followed by a sharp increase in 2022 [24]. In the United Kingdom, a comparable trend was noted, as influenza activity diminished during the restrictions associated with the COVID-19 pandemic. Following the lifting of these restrictions, influenza activity re-emerged in the 2022–2023 season [41].

A potential consequence of interventions to stop the transmission of infections in the community (NPIs) has been a phenomenon known as “immunity debt”, which refers to a reduction in population immunity due to the lack of exposure to common pathogens during a pandemic. The concept of immunity debt explains the increase in respiratory infections after the COVID-19 pandemic [42,43]. This phenomenon has been observed in various countries around the world. A study of a cohort of 1.79 million Danish children (0–17 years) over 10 years (2012–2022) revealed the impact of the COVID-19 pandemic and subsequent NPIs on the incidence of respiratory syncytial virus (RSV), influenza, and pneumococcus. Concerning influenza, there were 122 cases per 100,000 person-years pre-lockdown, 23 during lockdown, and as many as 337 post-lockdown, giving an incidence rate ratio post- vs. pre-lockdown of 2.75 (95% CI: 2.67–2.83). The proportion of influenza cases linked to hospital contacts was similar before and after the lockdown. NPIs for COVID-19 not only limited the spread of the target virus but also significantly reduced the incidence of RSV, influenza, and pneumococcal cases. The subsequent lifting of restrictions resulted in a significant increase in these infections, likely due to immune debt from reduced pathogen circulation [44]. As large-scale NPIs drive the immune debt, our findings provide insight into public health policies that promote the use of these interventions in the future. This study highlights how the COVID-19 pandemic affected patterns of respiratory illness beyond the virus itself. Leung et al. reported post-pandemic immune debt contributing to the resurgence of influenza in the U.S. and England. At the same time, Cohen et al. examined different types of infections in France, reinforcing the idea that the reduced circulation of pathogens during the introduction of NPIs to reduce the transmission of infections in the community likely led to the resurgence of respiratory infections [43,45]. This situation may be linked to insufficient protective immunity due to extended periods of low exposure to a specific pathogen, which leaves a larger portion of the population vulnerable to the disease. Another factor could be the disruption of vaccination programs; routine vaccinations that are part of nationally planned immunization efforts declined during the COVID-19 pandemic, which has consequently increased the number of people susceptible to preventable diseases [21]. In South Korea, NPIs were widely implemented to mitigate the spread of COVID-19, and during the intervention there was a decrease in hospitalizations for influenza, pneumonia, asthma, and chronic obstructive pulmonary disease [46].

These findings also mirror the fact that the strictures implemented during the first waves of the COVID-19 pandemic impacted the epidemiology of infections, not just concerning the SARS-CoV-2 virus itself. It is valuable to apply the lessons learned during seasonal spikes in respiratory virus infections, such as RSV or influenza. Measures like lockdowns can be difficult to implement in a socially acceptable or feasible way over the long term. However, other strategies—such as regular hand washing, wearing face masks when necessary, limiting social contact, and self-isolating in case of potential infection—tend to be more acceptable. This is particularly important during the peak flu season. In addition to NPIs, vaccination stands out as one of the most effective preventive measures against influenza. It is crucial to promote and maintain high levels of influenza vaccine uptake, as the current vaccination rates in our country remain too low.

During the entire period, in Poland, 1146 hospitalized patients died, accounting for 2.9% of all hospitalized patients due to influenza. In the pre-pandemic period, the mortality rate was 3.4%, and during the pandemic, it decreased to 2.1%. These values are in line with international data—in-hospital fatality in Japan during the 2017–2020 period was 2.8% (2.2% in 2017, 3.4% in 2018, 3.0% in 2019, and 2.3% in 2020) [47]. Between 2016 and 2021, 5.8% of people hospitalized with influenza in Spain died. This percentage nearly doubled to 10.8% between 2020 and 2021 [23]. In England, 7% of all hospital admissions resulted in a death between 2016 and 2020. During this period, the percentage of admissions that ended in death declined each year, dropping from 11% in the 2016–2017 season to 6% in the 2019–2020 season [34]. The highest number of deaths was in the 65+ years group. The case fatality ratio for this group in the pre-pandemic period ranged from 9.1% (2018) to 16.0% (2020) and remained at 16.0% during the pandemic. In contrast, the fewest deaths were recorded in the 0–4 and 5–19 age groups. It should also be noted that, according to studies by other authors, the highest number of deaths was observed among the oldest age groups. In the Japanese population, the 2017–2020 case fatality ratio for patients aged 70 and older hospitalized for influenza was 5.2% [47]. Before the COVID-19 pandemic in Italy, the majority of hospital deaths were among the oldest age group, while the least affected group were children aged 5–14 [29]. In the United States, the percentage of hospitalized patients who died in the hospital varied between 2.2% during 2021–22 and 3.5% in 2013–14. Among these patients, adults, particularly those aged 65 years and older, had the highest rates of in-hospital deaths, with percentages ranging from 3.3% in 2011–12 to 4.8% in 2013–14 [31]. A study conducted in the Netherlands from 2011 to 2020 estimated a 20% increase in the risk of death for each decade of life, implying a doubling of risk every five years [48]. In Wales, before the pandemic, annual incidence rates per 100,000 population for deaths attributable to influenza ranged from 0.6 (2015) to 2.3 (2018), before falling to 0.7 and 0.0 deaths in 2020 and 2021, respectively [22]. Other studies have also noted a decrease in mortality during a pandemic. This reduction may be attributed to a combination of lower hospitalization rates, changes in the organization of the healthcare system, and possible variations in the pathogenicity of the dominant influenza virus strains from year to year [49,50,51].

Influenza vaccination coverage in Poland remained very low and relatively stable throughout the analyzed period, with the percentage of people vaccinated against influenza not exceeding 3.4%. In the pre-pandemic years, vaccination rates varied from 2.2% in 2016 to 2.6% in 2018 and 2019. The vaccination rate slightly increased during the pandemic, reaching 3.4% in 2021. Influenza vaccination is an important tool for mitigating morbidity and mortality associated with influenza infections and has shown benefits even when patients are hospitalized for influenza. Studies have shown that vaccinated, hospitalized adult patients have a 26–59% lower risk of admission to an intensive care unit and a 31% lower risk of death compared to unvaccinated patients [31,52,53]. Vaccinations effectively reduce the spread of both influenza and COVID-19 infections. Influenza vaccination significantly reduced the risk of hospitalization and in-hospital mortality, especially in the elderly population [54,55,56]. Other systematic reviews and meta-analyses have shown that the types of influenza vaccines might be related to better protection against influenza-like illness, influenza-related hospitalizations, all-cause hospitalizations, or influenza-associated clinical complications [57,58].

The strengths of our study include its nationwide scope, encompassing the entire population of Poland, which ensured comprehensive data collection. However, several limitations should be considered when interpreting the results. The study relied on data from the Nationwide General Hospital Morbidity Study, but the reliability and consistency of the reported data were not specifically evaluated. Nevertheless, the legal obligation to report all hospitalizations and regulations governing data processing in official statistics suggest high data reliability. The analysis depended on the accuracy of medical coding, which poses challenges in monitoring influenza and flu-like syndromes due to nonspecific symptoms and the lack of routine laboratory testing. Consequently, incomplete or missing diagnosis codes in medical records may have led to an underestimation of hospital admissions and in-hospital mortality. Future research on influenza in Poland could explore long-term trends in the epidemiological landscape and the burden on the healthcare system. Additionally, other statistical methods, such as joinpoint regression, could be considered a valuable tool for analyzing changes in trends over time. Furthermore, although this study focused exclusively on influenza and did not include other respiratory pathogens, incorporating them could provide a more comprehensive picture of the epidemiological situation. Finally, reliance on national registries excluded detailed clinical assessments, potentially masking differences in infection severity. Regarding the interpretation of results, it is also important to note that immune debt may be influenced by multiple factors, including but not limited to NPIs. The continued monitoring of these trends and their implications for health system will help improve preparedness for future respiratory disease epidemics and optimize healthcare resource allocation.

## 5. Conclusions

Data from the NGHMS on influenza-related hospitalizations provide valuable information on the burden of influenza in Poland. Conducting analyses on hospitalizations and in-hospital mortality is crucial in providing data to support the optimization of planned and already implemented prevention strategies.

Our study demonstrates that the highest hospitalization rates per 100,000 population were observed among children under 5 years of age, while the highest in-hospital mortality rates were recorded in patients aged 65 years and older, both in the pre-pandemic period and during the COVID-19 pandemic. These findings point to the need to strengthen primary prevention strategies, including universal influenza vaccination and targeted public health education initiatives, to reduce the burden of severe influenza, especially in high-risk patient groups.

Our findings have the potential to influence health policy-making at national and local levels, including the development of tailored vaccination strategies, and to support operational management decisions, including planning and the allocation of hospital beds during a pandemic. Additionally, they emphasize the importance of enhancing the role of primary care in influenza management through proactive patient education and routine immunization. A comprehensive approach combining widespread vaccination, strong primary healthcare involvement, and public health interventions in the area of health promotion and disease prevention is key to minimizing influenza-related morbidity, hospitalizations due to severe forms of the disease, and the overall strain on the healthcare system.

## Figures and Tables

**Table 1:** Age and sex description of hospitalized patients in pre-pandemic and COVID-19 pandemic periods (* statistically significant, *p* < 0.001).

Gender/Age Groups	Pre-Pandemic Period		COVID-19 Pandemic Period	
	1 January 2016–3 March 2020		4 March 2020–31 December 2022	
	N	%	N	%
Men	13,484	100%	7922	100%
0–4 *	4890	36.3%	3588	45.3%
5–19 *	4311	32.0%	2874	36.3%
20–64 *	2225	16.5%	774	9.8%
≥65 *	2058	15.3%	685	8.6%
Average age (SD) *	22.8 (28.0)		14.9 (22.9)	
Women	11,639	100.0%	6558	100.0%
0–4 *	4117	35.4%	2922	44.6%
5–19 *	3314	28.5%	2221	33.9%
20–64 *	1982	17.0%	662	10.1%
≥65 *	2226	19.1%	753	11.5%
Average age (SD) *	26.0 (30.3)		17.1 (25.5)	
Total	25,123	100.0%	14,480	100.0%
0–4 *	9007	35.9%	6511	45.0%
5–19 *	7625	30.4%	5095	35.2%
20–64 *	4207	16.7%	1436	9.9%
≥65 *	4284	17.1%	1438	9.9%
Average age (SD) *	24.3 (29.2)		15.9 (24.2)	

**Table 2:** Number of hospitalization cases (N) and hospitalization rate per 100,000 population (rate) by age group.

		0–4 years	5–19 years	20–64 years	≥65 years	Total
2016	N	1293	1320	1062	554	4229
	rate	68.7	22.7	4.3	9.0	11.0
2017	N	991	701	523	822	3037
	rate	52.5	12.1	2.1	12.8	7.9
2018	N	1666	1654	1052	1314	5686
	rate	87.4	28.6	4.4	19.9	14.8
2019	N	2611	1775	1094	1103	6583
	rate	136.4	30.8	4.6	16.1	17.1
2020	N	3218	2804	1085	1006	8113
	rate	168.3	48.6	4.6	14.3	21.1
2021	N	297	64	55	70	486
	rate	15.8	1.1	0.2	1.0	1.3
2022	N	5441	4401	771	857	11,470
	rate	301.0	75.1	3.4	11.8	30.3

**Table 3:** In-hospital deaths (N; %) and case fatality ratio (CRF) in hospitalized influenza patients by age group.

Age Group		0–4 years	5–19 years	20–64 years	≥65 years	Total
2016	N	4	3	75	84	166
	%	2.4%	1.8%	45.2%	50.6%	100.0%
	CFR	0.31	0.23	7.06	15.16	3.9
2017	N	0	0	19	83	102
	%	0.0%	0.0%	18.6%	81.4%	100.0%
	CFR	0.00	0.00	3.63	10.10	3.4
2018	N	1	2	56	119	178
	%	0.6%	1.1%	31.5%	66.9%	100.0%
	CFR	0.06	0.12	5.32	9.06	3.1
2019	N	4	4	113	176	297
	%	1.3%	1.3%	38.0%	59.3%	100.0%
	CFR	0.15	0.23	10.33	15.96	4.5
2020	N	3	6	45	161	215
	%	1.4%	2.8%	20.9%	74.9%	100.0%
	CFR	0.09	0.21	4.15	16.0	2.7
2021	N	0	0	3	11	14
	%	0.0%	0.0%	21.4%	78.6%	100.0%
	CFR	0.00	0.00	5.45	15.71	2.9
2022	N	0	2	33	139	174
	%	0.0%	1.1%	19.0%	79.9%	0.0%
	CFR	0.00	0.05	4.28	16.22	1.5

**Table 4:** Description of influenza epidemiology in Poland (2016–2022).

	2016	2017	2018	2019	2020	2021	2022
All influenza cases	4,316,823	5,043,491	5,239,293	4,790,033	3,160,711	2,973,793	4,703,128
Incidence per 100,000 population	11,233.9	13,126.5	13,639.3	12,478.4	8240.9	7792.5	12,433.1
Number of people vaccinated against influenza	857,029	945,869	1,009,285	1,013,706	1,046,633	1,293,653	1,107,351
Percentage of people vaccinated against influenza	2.2%	2.5%	2.6%	2.6%	2.7%	3.4%	2.9%

## Data Availability

The datasets supporting the conclusions of this article are available in the National Institute of Public Health NIH—National Research Institute (NIPH NIH-NRI) repository and can be made available to researchers in accordance with legal restrictions. Researchers may apply for access to data from public administrative registries in Poland, such as NGHMS. The application must clearly specify the requested data and define the research objectives. For inquiries and access requests, please contact NIPH NIH-NRI at pzh@pzh.gov.pl.
